# Evaluating the Prognostic Relevance of Pre-Treatment Epstein–Barr Virus Levels in Non-Endemic Pediatric Nasopharyngeal Carcinoma

**DOI:** 10.3390/cancers18172800

**Published:** 2026-08-28

**Authors:** Ahmed Farrag, Yanbo Yang, Jin Piao, Lindsay Younis, Hans-Joachim Wagner, Hans Christiansen, Tristan Römer, Allison Poore, Christopher Szot, Nadia Thibeau, Sue S. Yom, Benjamin A. Pinsky, Quynh-Thu Le, Junne Kamihara, David T. Ting, Theodore W. Laetsch, Kenneth S. Chen, Carlos Rodriguez-Galindo, Randall T. Hayden, Udo Kontny, Robyn D. Gartrell

**Affiliations:** 1 Division of Pediatric Hematology, Oncology and Stem Cell Transplantation, Medical Faculty, RWTH Aachen University, 52074 Aachen, Germany; ahmedfarrag@aun.edu.eg (A.F.); troemer@ukaachen.de (T.R.); ukontny@ukaachen.de (U.K.); 2 Center for Integrative Oncology Aachen Bonn Cologne Düsseldorf, 52074 Aachen, Germany; 3 Pediatric Oncology Department, South Egypt Cancer Institute, Assiut University, Assiut 71515, Egypt; 4 Division of Pediatric Hematology and Oncology, Department of Pediatrics, Children’s Hospital of Central Switzerland, 6004 Lucerne, Switzerland; 5 Division of Pediatric Oncology, Department of Oncology, Johns Hopkins University School of Medicine, Baltimore, MD 21287, USA; yyang304@jh.edu; 6 Department of Population and Public Health Sciences, University of Southern California, Los Angeles, CA 90032, USA; jpiao@childrensoncologygroup.org; 7 Children’s Oncology Group, Monrovia, CA 91016, USA; lyounis@childrensoncologygroup.org (L.Y.); nthibeau@childrensoncologygroup.org (N.T.); 8 Department of Pediatric Hematology and Oncology, Justus Liebig University Giessen, 35392 Giessen, Germany; hans-jwagner@web.de; 9 Department of Radiotherapy and Radiation Oncology, Hannover Medical School, 30625 Hannover, Germany; christiansen.hans@mh-hannover.de; 10 Biospecimen Research Group, Frederick National Laboratory for Cancer Research, Frederick, MD 21702, USA; younkinsa@gmail.com (A.P.); christopher.szot@nih.gov (C.S.); 11 Department of Radiation Oncology, University of California San Francisco, San Francisco, CA 94115, USA; sue.yom@ucsf.edu; 12 Department of Pathology, Stanford University School of Medicine, Stanford, CA 94305, USA; bpinsky@stanford.edu; 13 Department of Radiation Oncology, Stanford University, Stanford, CA 94304, USA; qle@stanford.edu; 14 Dana-Farber/Boston Children’s Cancer and Blood Disorders Center, Harvard Medical School, Boston, MA 02115, USA; junne_kamihara@dfci.harvard.edu; 15 Krantz Family Center for Cancer Research, Mass General Brigham Cancer Institute, Harvard Medical School, Boston, MA 02129, USA; dting1@mgh.harvard.edu; 16 Division of Oncology, Center for Childhood Cancer Research and Cancer Immunotherapy Program, Children’s Hospital of Philadelphia, Philadelphia, PA 19104, USA; laetscht@chop.edu; 17 Department of Pediatrics, UT Southwestern Medical Center, Dallas, TX 75390, USA; kenneth.chen@utsouthwestern.edu; 18 Department of Oncology and Global Pediatric Medicine, St. Jude Children’s Research Hospital, Memphis, TN 38105, USA; carlos.rodriguez-galindo@stjude.org; 19 Department of Pathology, St. Jude Children’s Research Hospital, Memphis, TN 38105, USA; randall.hayden@stjude.org; 20 Departments of Pediatrics and Radiation Oncology, Johns Hopkins University School of Medicine, Baltimore, MD 21287, USA

**Keywords:** pediatric nasopharyngeal carcinoma, nasopharyngeal carcinoma, pediatric cancers, rare cancers, head and neck cancers, oral cancers, Epstein–Barr virus, biomarkers

## Abstract

**Background/Objectives:** Pediatric nasopharyngeal carcinoma (NPC) is a very rare childhood cancer strongly associated with Epstein–Barr virus (EBV) infection. We investigated the prognostic value of EBV DNA on staging and outcome in pediatric NPC from two large study centers, the Children’s Oncology Group (COG) in North America and the German Society of Pediatric Oncology and Hematology (GPOH) in Europe. **Methods:** Samples collected from NPC patients treated on the COG study ARAR0331 between 2006 and 2012 and the GPOH NPC protocol between 2003 and 2021 were retrospectively analyzed for the level of available plasma (P-EBV) or whole-blood EBV DNA (WB-EBV), both pre-treatment and post-induction chemotherapy. Patients were dichotomized into high and low groups based on the median pre-treatment EBV value. **Results:** Pre-treatment EBV DNA levels from 102 patients (50 and 23 P-EBV DNA from the COG and GPOH, respectively, and 29 WB-EBV from GPOH) and post-induction EBV DNA levels from 61 patients (31 and 12 P-EBV DNA from the COG and GPOH, respectively, and 18 WB-EBV DNA from GPOH) were evaluated. Patient characteristics, including age and disease stage, were not associated with high and low P-EBV values in any cohort. Disease stage correlated with high EBV levels in the WB-EBV GPOH cohort (*p* = 0.014). Pre-treatment P-EBV and WB-EBV levels showed no significant association with 5-year event-free survival (EFS: COG *p* = 0.65, GPOH P-EBV: *p* = 0.08, GPOH WB-EBV: *p* = 0.75) or 5-year overall survival (OS: COG *p* = 0.90, GPOH P-EBV *p* = 0.17, GPOH WB-EBV *p* = 0.19). **Conclusions:** Our study could not establish a significant correlation between outcome and pre-treatment EBV DNA in pediatric NPC patients from non-endemic areas.

## Simple Summary

Epstein–Barr virus (EBV) DNA levels predict treatment response and outcomes in EBV-associated adult nasopharyngeal carcinoma (NPC) in both endemic and non-endemic regions, and in pediatric NPC in endemic regions. However, their prognostic value has not been studied in pediatric NPC in non-endemic regions. The Children’s Oncology Group (COG) and the German Society of Pediatric Oncology and Hematology (GPOH) collaborated to evaluate whether EBV DNA levels predict treatment response or outcomes in pediatric NPC. Non-endemic regions represented by COG included the United States, Canada and Australia; GPOH included Germany, Austria, Switzerland, and the Netherlands. We found that pre-treatment EBV DNA levels were not predictive of survival or treatment response in non-endemic pediatric NPC treated on COG and GPOH protocols. Because EBV DNA is used in the World Health Organization guidelines to prognosticate adult NPC at diagnosis and after treatment, our findings suggest that this approach requires further evaluation in children and adolescents with NPC outside endemic regions.

## 1. Introduction

Nasopharyngeal carcinoma (NPC) is relatively uncommon, with the highest incidence in adults in Southeast Asia, and in pediatric patients, it is often associated with Epstein–Barr virus (EBV) [1,2]. In North America, pediatric NPC patients are more commonly black and present more frequently with stage IV disease; it is considered very rare, with an incidence of 0.5 per million per year compared to 8.4 in adults [3]. Pediatric NPC has distinctive clinical and molecular features, as well as a relatively decreased mortality risk compared with adult NPC [4]. Risk factors for adult NPC include genetic factors, high consumption of salty foods, herbs, smoking, low socio-economic status, and, most importantly, infection with EBV [2,5,6,7,8].

In studies of adult NPC, the addition of pre-treatment EBV DNA levels as an adjunct biomarker for NPCs with anatomical staging provides more accurate prognostic information compared to TNM staging alone, potentially allowing for better treatment planning [9]. Prognostic nomograms have been built and validated for overall survival (OS) and disease-free survival (DFS) in NPC patients over 18 years of age with locoregionally advanced NPC, finding that high plasma EBV DNA (P-EBV) levels predict worse prognosis [10]. Pre-treatment P-EBV testing in adults has also been used for treatment monitoring, post-treatment surveillance for detecting relapse, and monitoring recurrent/metastatic disease [11]. EBV DNA levels in children in endemic regions have also been associated with prognosis; however, this has not been studied outside of these endemic regions in North America or Europe [12].

While multimodal chemoradiotherapy achieves favorable OS rates in children, the treatment is associated with significant toxicity [13]. The Children’s Oncology Group (COG) study ARAR0331 evaluated treatment for children with stage IIb to IV NPC using a protocol similar to adult therapy with cisplatin and fluorouracil, followed by concurrent chemoradiotherapy consisting of radiation with 2–3 additional cycles of cisplatin [14]. In ARAR0331, the 5-year event-free survival (EFS) rates for stages II, III, and IV were 100%, 82.8%, and 82.7%, respectively. Patients received 61.2 Gy of radiation if they showed a complete or partial response (PR) to induction chemotherapy and 71.2 Gy if they had stable disease according to Response Evaluation Criteria in Solid Tumors (RECIST) criteria. Their response showed that it is possible to achieve a dose reduction in radiation that would match the doses used by the German Pediatric Oncology and Hematology Society (GPOH). This has led to the current COG clinical trial ARAR2221, where dose-reduced radiation is determined by response after 3 cycles of chemoimmunotherapy using cisplatin, gemcitabine and nivolumab [13].

The NPC-GPOH-2003 study was a prospective multicenter study for patients with localized NPC up to the age of 25 years and enrolled 45 patients from Germany, Austria, Switzerland and the Netherlands from 2003 to 2010 [15]. The treatment plan consisted of three cycles of induction chemotherapy with cisplatin and 5-flurouracil, followed by concurrent chemoradiotherapy with cisplatin. The radiation dose to the primary tumor was 59.4 Gy and was reduced to 54 Gy for patients with complete response (CR) after induction. Chemoradiotherapy was followed by six months of maintenance therapy with Interferon-beta (IFNβ). The EFS and OS rates of the NPC-GPOH-2003 study cohort were 93.2% (95% CI, 80.3–97.7%) and 95.2% (95% CI, 82.3–98.8%), respectively, after a median follow-up of 85 months [16]. The NPC-GPOH-2003 study treatment protocol has been adopted as the standard of treatment for pediatric patients with NPC in Germany [2]. In 2023, GPOH opened the NPC-Nivo trial using the backbone of the NPC-2003 study with the addition of nivolumab to the induction chemotherapy with cisplatin and 5-fluorouracil, aiming to increase the percentage of patients with a complete response after induction therapy in order to reduce the dose of radiotherapy and potentially decrease the burden of late effects [17].

The interpretation of circulating EBV DNA depends importantly on the type of analyzed blood sample. P-EBV DNA predominantly represents cell-free viral DNA and, in EBV-associated NPC, is thought to originate largely from EBV-positive tumor cells, reflecting tumor burden and tumor-cell turnover. Accordingly, pre-treatment P-EBV DNA has been extensively investigated as a prognostic biomarker in NPC in endemic areas, especially in adults, and has been consistently associated with disease stage, recurrence, distant metastasis, and survival outcomes [18,19]. In contrast, EBV DNA measured in whole blood includes DNA derived from the cellular blood compartment, particularly EBV-infected circulating B lymphocytes, in addition to cell-free DNA, and therefore may reflect both the host EBV reservoir and tumor-associated EBV DNA. Consequently, EBV DNA concentrations measured in plasma and whole blood should not be considered quantitatively interchangeable, and differences in specimen type may have important implications for clinical interpretation. Although whole-blood EBV DNA has demonstrated diagnostic and prognostic utility, P-EBV DNA currently has a more established evidence base in NPC, particularly for pre-treatment risk stratification and treatment-response monitoring [18,20]. Nevertheless, plasma should not be regarded as universally superior; the optimal specimen depends on the clinical question, disease context, analytical assay, and pre-analytical procedures.

The prognostic significance of EBV DNA levels has been understudied in NPC patients under the age of 18, especially in North America and Europe, likely due to the very rare nature of this disease in these areas. Given the distinct biological characteristics in younger patients with NPC compared to adults, and the increasing use of EBV DNA quantification as a prognostic and predictive marker in adult NPC, it is important to determine if this biomarker operates similarly in pediatric patients from non-endemic regions. This study aimed to explore the prognostic value of pre-treatment EBV DNA levels in pediatric NPC patients treated in North America and Europe.

## 2. Materials and Methods

### 2.1. Patient Characteristics

The study was approved by the Johns Hopkins University School of Medicine Institutional Review Board (IRB) (IRB00420834) and the Ethics Committee of the Medical Faculty of the RWTH Aachen (EK 503/20). Written informed consent was obtained prior to participation from all patients treated within the included study protocols. Data from both male and female children and adolescents with NPC—treated in the COG study ARAR0331 from 2006 to 2012 in North America or treated or consulted within the GPOH network between 2003 and 2021 in Germany, Austria, the Netherlands, and Switzerland, and available at the NPC study center of the GPOH at Rheinisch-Westfälische Technische Hochschule (RWTH) Aachen University Hospital—were analyzed retrospectively. Parameters analyzed included the patient’s age, sex, stage of disease according to the American Joint Cancer Classification (AJCC) at the time of diagnosis, outcome, status, and the value of available P-EBV DNA or whole-blood EBV DNA (WB-EBV) pre-treatment and post-induction chemotherapy, as well as the radiological response to therapy using magnetic resonance imaging (MRI). Response to therapy data was collected at the end of induction chemotherapy, after chemoradiotherapy, and after interferon therapy, if available. Survival data from COG and GPOH was collected throughout therapy and for at least 5 years after completion.

### 2.2. Children’s Oncology Group (COG) Study ARAR0331

A total of 81 plasma specimens were included in the study from ARAR0331, comprising 50 specimens collected pre-treatment and 31 collected post-induction, with 20 having matched pre-treatment and post-induction specimens evaluated. Of the 31 patients with post-induction plasma specimens, only 27 were evaluable for response, as four patients did not have response data after chemoradiotherapy (2 patients came off protocol therapy at the end of induction, and response evaluation was not completed by the site for the other 2 patients). Histology included NPC World Health Organization (WHO) stage II or III and AJCC 5th edition stages IIb to IV in Stratum B. During the trial, plasma specimens were collected at diagnosis and after three 21-day cycles of induction chemotherapy.

### 2.3. German Society of Pediatric Oncology and Hematology (GPOH)

Samples were analyzed from two cohorts of patients. The first cohort consisted of 26 patients, including 22 patients from the NPC-GPOH-2003 study, which actively enrolled patients with non-metastatic NPC from January 2003 until December 2010 [15], as well as four patients with metastases treated according to the NPC-GPOH 2003 study protocol from 2011 to 2017. For centralized measurement of EBV DNA, plasma was used (“P-EBV-cohort”). The second cohort consisted of 34 patients treated according to the NPC-GPOH-2003 study protocol. Data were either obtained from patient samples which were provided to us as part of a consulting service (*n* = 17) (2011–2017) [16] or from patients who were enrolled into the NPC-2016 registry (*n* = 17) starting in October 2017. In contrast to the first cohort, this cohort included patients with both non-metastatic and metastatic disease. EBV DNA of the second cohort was centrally examined based on whole blood (“WB-EBV-cohort”). Of the 60 total patients included in the GPOH analysis, 52 had pre-treatment specimens and 30 had post-induction specimens, with 22 patients having matched pre-treatment and post-induction specimens evaluated. Data were collected in the period from January 2021 till December 2025, all centers were actively contacted for follow up; last date of follow up was December 2025.

### 2.4. EBV DNA Extraction, Processing and Analysis

#### 2.4.1. COG ARAR0331 Plasma Sample Processing and Droplet Digital PCR (ddPCR) Quantification

Plasma collections included 5 mL of blood in an EDTA tube, which were then processed via centrifugation. Plasma was drawn off at the collection site and stored at −80 °C to −70 °C until shipped on dry ice to the COG Biobank located at the Biopathology Center (BPC) at Nationwide Children’s Hospital. Biological samples were stored at −90 °C to −70 °C until molecular testing. Viral DNA was extracted from 200 μL of plasma using the QIAamp MinElute Virus Spin Kit (Qiagen, Valencia, CA, USA, 57704) according to the manufacturer’s instructions, performed manually. DNA was eluted in 50 μL of Buffer AVE.

EBV viral load was quantified using droplet digital PCR (ddPCR) targeting the BamHI-W fragment. The reference standard was an EBV standard from Exact Diagnostics (Bio-Rad, Hercules, CA, USA, EBVP100), calibrated against the 1st WHO International Standard for Epstein–Barr virus.

Primers and probe sequences were adapted from Miller et al. [21]. Forward primer: 5′-CCCAACACTCCACCACACC-3′; reverse primer: 5′-TCTTAGGAGCTGTCCGAGGG-3′; probe: 5′-CACACACTACACACACCCACCCGTCTC-3′. The ddPCR reactions were performed using the QX200 AutoDG System (Bio-Rad) with thermal cycling on a C1000 Touch Thermal Cycler (Bio-Rad).

The threshold determination and absolute quantification were performed using the ddpcRquant package in R (4.4.1) [22]. Plasma negative samples were used to define the threshold for each plate; wells with <10,000 accepted droplets were excluded from analysis. The droplet fluorescence amplitude plots for each well were visually inspected by two independent investigators, and any conflicts were resolved by a third investigator. Wells with only droplets below the threshold or with intermediate fluorescence overlapping the negative population were classified as negative, and subjects were classified as negative if the majority of the wells were classified as negative. Known standard samples with defined viral titers (5 × 10^2^ to 5 × 10^5^ IU/mL) were used to construct a calibration curve by fitting a log-log linear regression model of IU/mL against measured concentration. Outliers within each sample measurement were identified and excluded using the 1.5 × IQR method.

#### 2.4.2. GPOH EBV DNA Specimen Processing and Quantification of EBV DNA

Plasma samples:

EBV-PCR plasma titers were analyzed in the laboratory of H.-J.W. at the Medical University of Lübeck (2003 to May 2008) and University Hospital of Giessen (June 2008–2011) as described previously [23]. DNA from 200 microliters of EDTA-plasma of each patient was isolated using the QIAamp Blood Mini Kit (QIAGEN, Valencia, CA, USA) as recommended by the manufacturer. The concentration and purity of the obtained DNA were analyzed by a spectrophotometer (Ultrospec 2000, Pharmacia Biotech, Freiburg, Germany). For detection and quantification of EBV-specific sequences, an EBV-specific real-time quantitative polymerase chain reaction assay was developed using 5′ nuclease PCR technology and the ABI PRISM 7700 Sequence Detection System (PE Applied Biosystems, Foster City, CA, USA) [24].

A set of primers and probes specific for the BAM HI-K region of EBV, as well as one set of primers and a probe specific for the CRP region as an internal control, were designed using sequence data from the GenBank Sequence Database (National Library of Medicine, National Institute of Health, Bethesda, MD, USA) and the Primer Express Software (3.0.1, PE Applied Biosystems) [23].

The sequences for the primers and probes were as follows: BAM HI-K forward primer: 5′-CCG GTG TGT TCG TAT ATG GAG-3′; BAM HI-K reverse primer: 5′-GGG AGA CGA CTC AAT GGT GTA-3′; BAM HI-K probe: 5′VIC-TGC CCT TGC TAT TCC ACA ATG TCG TCT T-TAMRA 3′; CRP forward primer: 5′-CTT GAC CAG CCT CTC TCA TGC-3′; CRP reverse primer: 5′-TGC AGT CTT AGA CCC CAC CC-3′; CRP probe: 5′FAM-TTT GGC CAG ACA GGT AAG GGC CAC C-TAMRA-3′. DNA preparations from an equivalent of 40 microliters of plasma were analyzed per reaction.

PCR amplification was performed using TaqMan Polymerase as described previously [23]. Real-time measurements were taken, and a Ct value was calculated for each sample. The EBV-positive Burkitt’s lymphoma cell line Namalwa (American Type Culture Collection [ATCC], Manassas, VA, USA, CRL-1432) was used as a standard for quantification of EBV DNA. The detection limit was 5 EBV copies per analysis.

Whole blood samples:

Whole-blood EBV DNA was analyzed centrally at the Institute of Virology of Hannover Medical School using quantitative-PCR with a modified method originally described by Engelmann et al. [25]. In brief, DNA was isolated from 1.2 mL of peripheral blood. For automated purification of nucleic acids, a robotic workstation (QIAcube, Qiagen GmbH, Hilden, Germany) with the QIAamp DNA Blood Kit Cat. No 51104 (Qiagen) was used. Amplification of purified nucleic acids was done using the Quantitec Multiplex-PCR Kit # Cat. No. 204545 from Qiagen with the following primers: EBV TQ1: 5′ TTC CAC CGC CGA GAT GAT (0.5 µM); EBV TQ2: 5′ TCG TAA GAC GGA GAG ATG TCC AT (0.5 µM); and the TaqMan EBV probe: 5′FAM- ACT TGC GCC CTC CTC TTG AAT CG–TAMRA (0.4 µM). Amplification was performed on an Applied Biosystems (ABI) 3130 Genetic Analyzer under the following conditions: Activation of the HotStar Taq DNA Polymerase for 15 min at 95 °C, followed by 45 cycles of denaturation for 20 s at 95 °C and an annealing and elongation step for 1 min at 60 °C.

A three-point calibration with three defined standards was performed in each PCR run for quantification. The linear range was between 1600 up to 2.5 × 10^9^ copies/mL, equaling 3200 up to 5 × 10^9^ IU/mL, and the limit of detection was 250 copies/mL (500 IU/mL). Therefore, negative results had a viral load of less than 500 IU/mL. Since the linear range of the method started at 3200 IU/mL, numerical values below 3200 IU/mL were outside the evaluable range and could not be evaluated quantitatively, but we collected these raw numerical values so that a median could be created, and we processed the data similarly to the COG cohort for purpose of comparison. The results were expressed as IU/mL, which had been converted from the number of copies of the EBV genome per milliliter of blood.

### 2.5. Statistical Analysis

#### 2.5.1. COG ARAR0331

The primary objective was to quantify P-EBV in pediatric patients with NPC enrolled in ARAR0331 and to determine whether pre-treatment P-EBV levels correlated with EFS. Patients with available pre-treatment P-EBV quantitative data were dichotomized at the cohort median into low (<median) and high (>median) P-EBV groups. EFS was defined as the time from patient enrollment until disease relapse/progression, diagnosis of a second malignant neoplasm, death from any cause, or last follow-up, whichever occurred first. OS was defined as the time from enrollment until death from any cause or last follow-up, whichever came first. The EFS distribution was estimated using the Kaplan-Meier method, with 95% confidence intervals computed using the log-log transformation. Differences in EFS or OS between P-EBV groups were assessed using the log-rank test. Cox proportional hazards models were used to evaluate the association between P-EBV group and EFS/OS, adjusting for relevant covariates. Outcome data for patients treated on COG ARAR0331 current up to 30 June 2024 were used in the analysis. Percent change was calculated for these patients by subtracting the pre-treatment IU/mL from the post-induction value and dividing by the pre-treatment value. All analyses were performed using R version 4.3.1.

#### 2.5.2. GPOH

All statistical calculations were done using SPSS (statistical package for the social sciences; SPSS Inc., Chicago, IL, USA) version 26. Quantitative data were statistically described in terms of mean ± SD or median (range) when not normally distributed. Qualitative data were statistically described in terms of frequencies (number of cases) and relative frequencies (percentages) when appropriate. Comparison of quantitative variables was done using Student’s *t* test for normally distributed data and the Mann–Whitney U test for non-normally distributed data. For comparing categorical data, the chi-square (χ^2^) test was performed. Fisher’s exact test was used instead when the expected frequency was less than 5. Kaplan–Meier’s method with log-rank test was used for OS and EFS analysis. The hazard ratio (HR) with 95% confidence interval (CI) and Cox regression analysis were calculated to determine significant factors associated with mortality, when applicable. The *p*-value was always 2-tailed, set significant at the 0.05 level.

Patients were dichotomized into high and low groups based on the median pre-treatment EBV level. Patients with values ≤ median were classified as low; those with values > median as high. The percent change was calculated for these patients by subtracting the pre-treatment average value from those at post-induction chemotherapy and dividing again by the pre-treatment sample. This created a negative percentage for any decrease and a positive percentage for any increase in the value of EBV DNA. If the patient had the same EBV result pre-treatment and post-induction, the percent change was considered “No change”. The P-EBV values were expressed in copies/mL, and as quantification was performed years before without an appropriate conversion factor, the copies/mL could not be transformed to IU/mL. No data were available on the patients’ race or ethnicity within the GPOH group. For statistical purposes, all negative EBV-PCR results with no available exact values were considered to be zero.

## 3. Results

### 3.1. Distribution of Pre-Treatment EBV DNA Levels Is Similar Between COG and GPOH Cohorts

#### 3.1.1. Distribution of Pre-Treatment P-EBV DNA Levels Is Not Associated with Age or Stage for Patients Treated on COG ARAR0331

**Figure 1:**
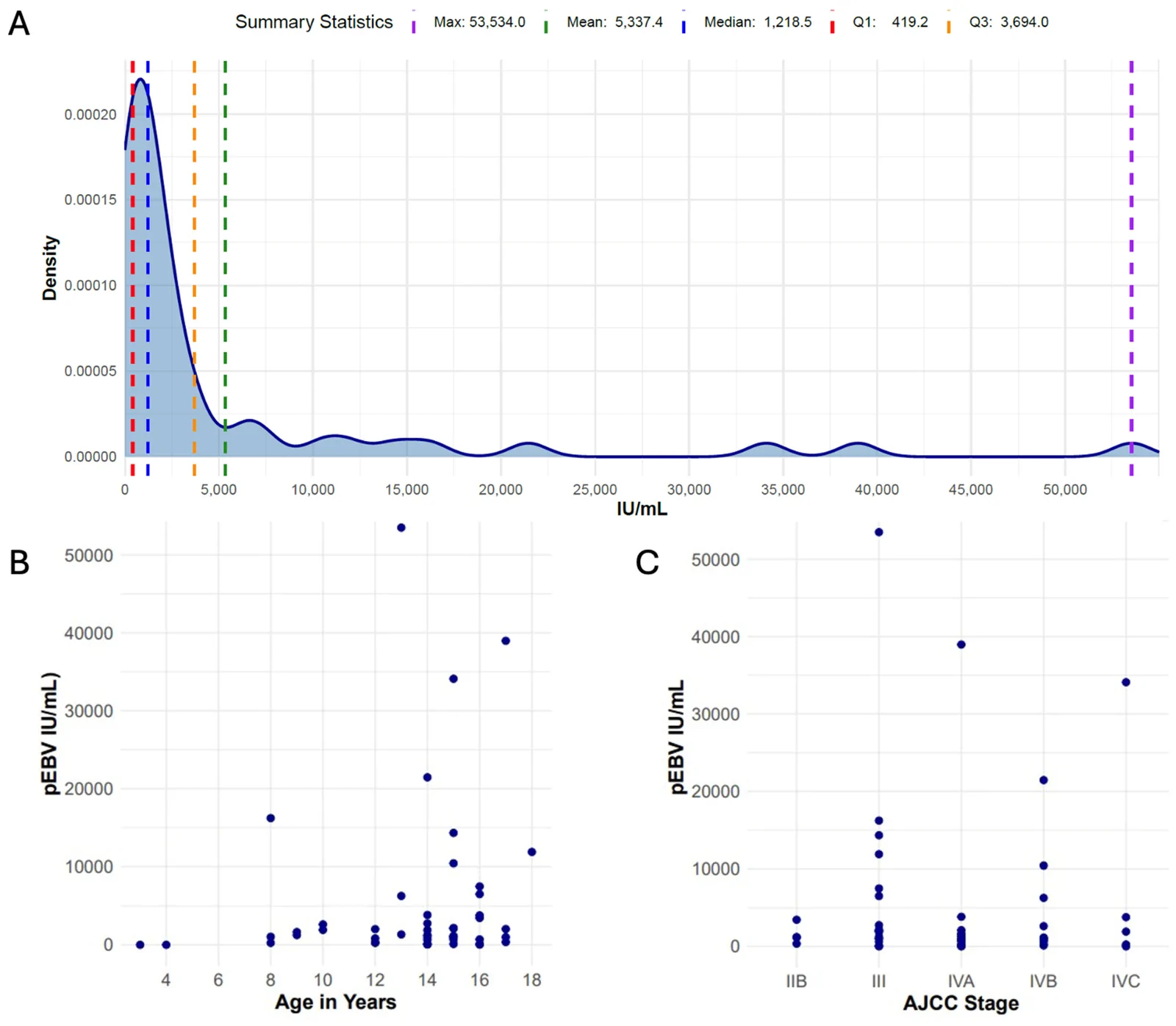
Distribution of P-EBV (IU/mL) pre-treatment for patients treated on COG ARAR0331 (**A**) Distribution of all patients showing summary statistics in colored lines including max (purple), mean (green), median (blue), first quartile (Q1, red) and 3rd quartile (Q3, orange). (**B**) Distribution of P-EBV (IU/mL) by age at diagnosis in years and (**C**) Distribution of pre-treatment P-EBV (IU/mL) by stage using AJCC 5th edition staging.

A total of 50 pediatric patients with NPC from COG ARAR0331 were included in the analysis of pre-treatment P-EBV levels. The distribution of P-EBV was plotted with a mean pre-treatment P-EBV level of 5337.4 IU/mL (SD = 10,630.5), a median of 1218.5 IU/mL and a 3rd quartile of 3694 IU/mL (Figure 1A). The distribution of P-EBV across ages in years (Figure 1B) and across stages (Figure 1C) was also plotted. Among all 50 patients, 48 had a positive P-EBV DNA value (96%). Of the two patients with negative P-EBV, one had a known positive EBER on pathology, while the other patient’s pathology report did not document EBER testing. Patient characteristics for the P-EBV cohort are shown in Table 1. There was an even split among black and white races (both 44%), and a majority of the patients were non-Hispanic (94%). Most patients in the study had stage 3 disease (38%), followed by stage IVA (24%) and stage IVB (20%) with few patients in stage II (8%) or stage IVC (10%) (Table 1). Patient characteristics were evaluated, showing no difference between high and low P-EBV when dichotomized by median (Supplemental Table S1).

#### 3.1.2. Distribution of Pre-Treatment P-EBV DNA Levels Is Not Associated with Age or Stage for Patients from GPOH Cohorts While WB-EBV DNA Correlates with Stage Only

**Figure 2:**
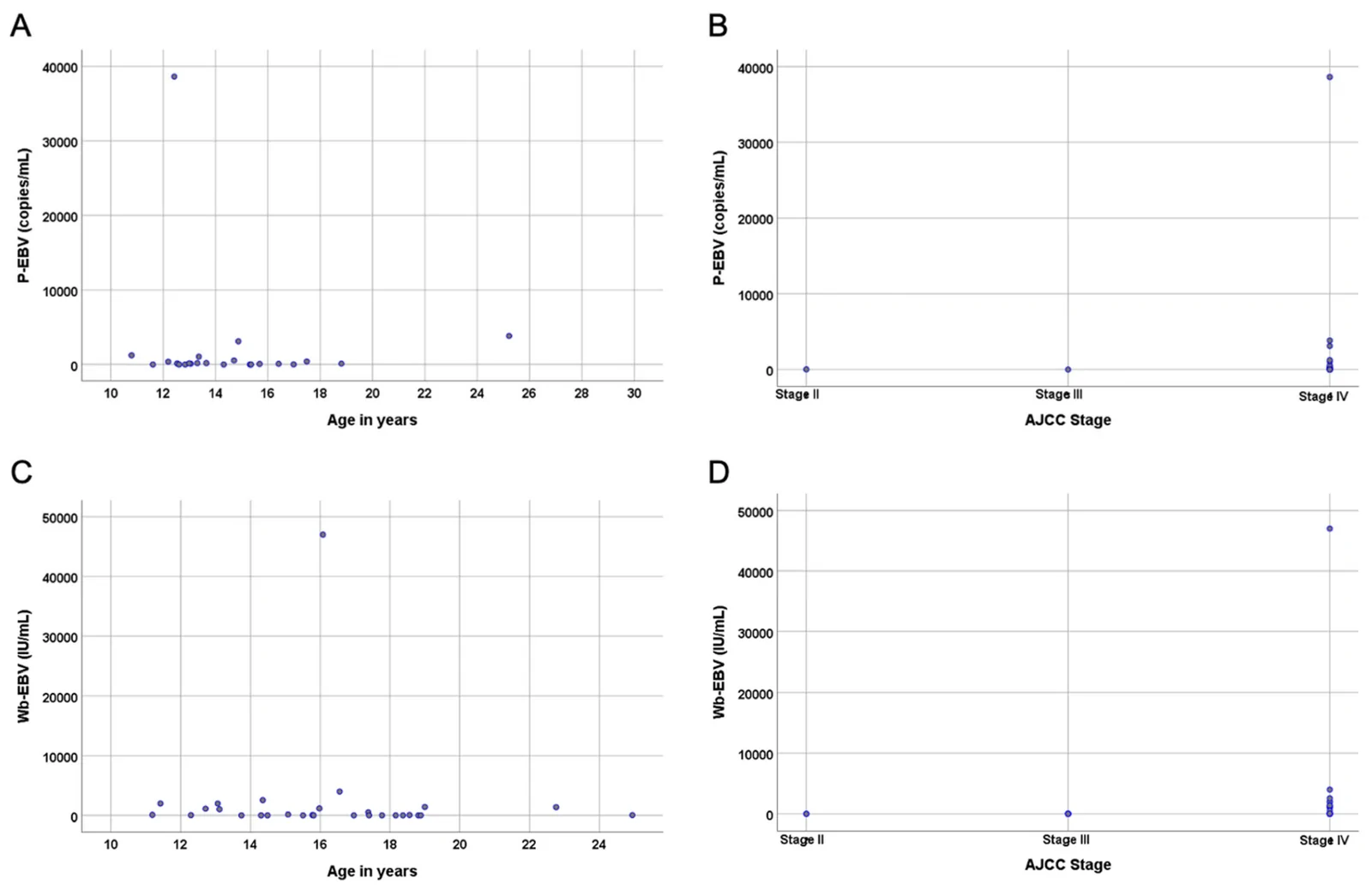
Distribution of P-EBV and WB-EBV at diagnosis by age and stage for patients with NPC in GPOH. (**A**) Scatter plot diagram showing the correlation between P-EBV (copies/mL) and the age of the studied patients with NPC in GPOH. (**B**) Scatter plot diagram showing the correlation between P-EBV (copies/mL) and stage of disease in patients with NPC in GPOH. (**C**) Scatter plot diagram showing the correlation between WB-EBV (IU/mL) and the age of the studied patients with NPC in GPOH. (**D**) Scatter plot diagram showing the correlation between WB-EBV (IU/mL) and stage of disease in patients with NPC in GPOH.

Patient summary statistics for all patients included in the GPOH study analysis of P-EBV and WB-EBV DNA are shown in Table 1. For pre-treatment P-EBV analysis, a total of 23 pediatric patients with NPC were included. The distribution of pre-treatment P-EBV was plotted, with a mean P-EBV level of 2182 copies/mL (SD = 8004.43), a median of 135 copies/mL (range: 0:38,619 copies/mL) and a 3rd quartile of 539 copies/mL. Among all 23 patients, 17 had a positive P-EBV DNA value (74%). The distribution of P-EBV across ages in years (Figure 2A) and across stages (Figure 2B) was also plotted. Patients’ characteristics comparing high and low P-EBV using the median as a cut off showed no significant difference between high and low P-EBV groups (Supplemental Table S2).

For pre-treatment WB-EBV DNA analysis, a total of 29 pediatric patients were included; 17 of them (59%) had a positive WB-EBV DNA value. The distribution of pre-treatment WB-EBV was plotted, with a mean pre-treatment WB-EBV level of 2225 IU/mL (SD = 8667.63) and a median of 53 IU/mL (range 0:47,000 IU/mL). The distribution of WB-EBV DNA across ages in years (Figure 2C) and across stages (Figure 2D) was also plotted. Patient summary statistics for the pre-treatment WB-EBV cohort are shown in Supplemental Table S3. Patient characteristics evaluated showed no significant difference between high and low WB-EBV, except for disease stage. Thirteen out of 14 patients from the high WB-EBV group had stage IV disease (92.9%), versus only 7 of the 15 patients (46.7%) in the low WB-EBV group (*p* = 0.014). Of the 6 patients with metastatic disease, 5 were in the high WB-EBV group vs. only 1 patient in the low WB-EBV group (*p* = 0.080).

### 3.2. Association of Pre-Treatment EBV DNA Levels with Survival in Pediatric NPC Patients Enrolled in COG and GPOH Studies

#### 3.2.1. Association of Pre-Treatment EBV DNA Levels with EFS and OS Among Patients Treated on COG ARAR0331

**Figure 3:**
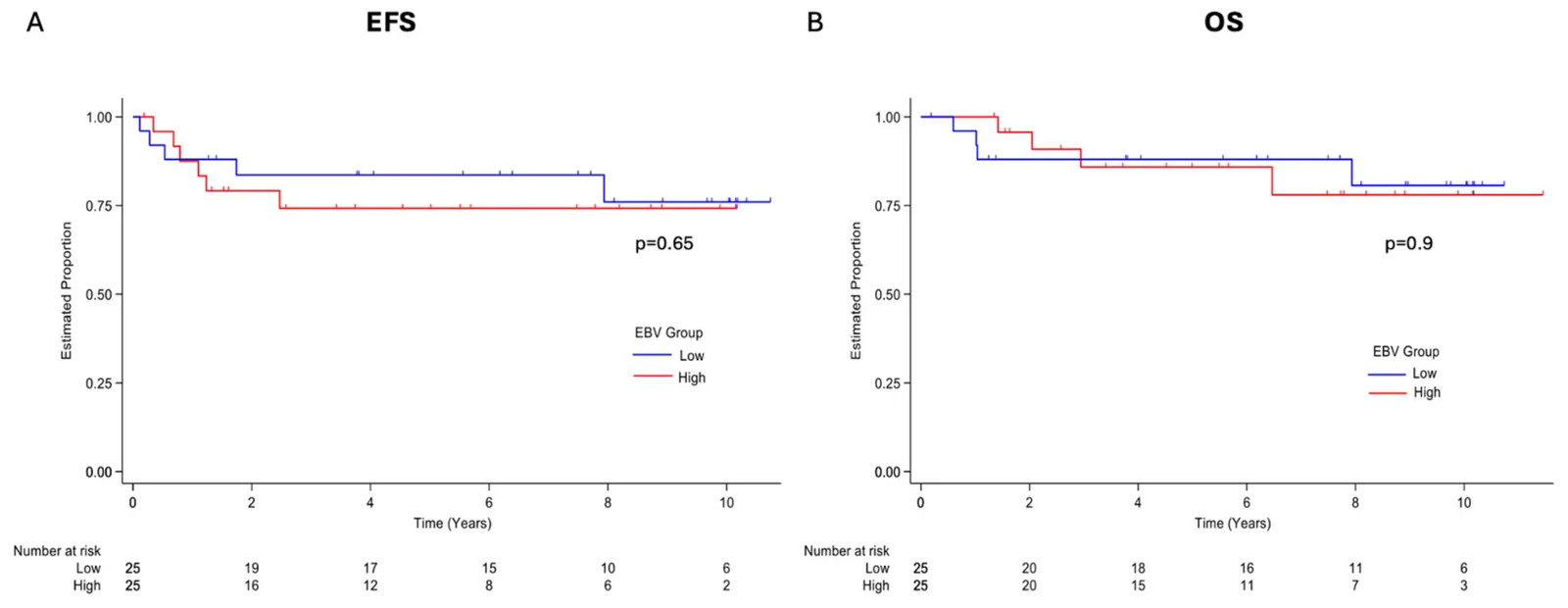
Survival analysis from 50 patients treated on ARAR0331 comparing high and low pre-treatment P-EBV DNA levels. (**A**) Event-free survival (EFS) is 83.6% (95% CI: 61.9–93.5%) for the low P-group and 74.2% (95% CI: 51.2–87.6%) for the high group (*p* = 0.65, HR = 1.32, 95% CI: 0.40–4.35) and (**B**) overall survival (OS) is 88% (95% CI: 67.3–96%) for the low P-EBV group and 85.8% (95% CI: 62.1–95.2%) for the high group (*p* = 0.9, HR = 1.1, 95% CI: 0.27–4.41).

Eleven patients experienced events during follow-up (five in the low group, six in the high group). There was no significant difference between high and low P-EBV when evaluating 5-year event-free survival (EFS, Figure 3A) or 5-year overall survival (OS, Figure 3B). EFS was 83.6% (95% CI: 61.9–93.5%) for the low P-EBV group and 74.2% (95% CI: 51.2–87.6%) for the high group (*p* = 0.65, HR = 1.32, 95% CI: 0.40–4.35) (Figure 3A). OS was 88% (95% CI: 67.3–96%) for the low P-EBV group and 85.8% (95% CI: 62.1–95.2%) for the high group (*p* = 0.9, HR = 1.1, 95% CI: 0.27–4.41) (Figure 3B). In multivariate Cox modeling adjusted for age, race, and grouped stage, pre-treatment P-EBV group was not significantly associated with EFS (HR = 1.12, 95% CI: 0.32–3.97; *p* = 0.86) (Supplemental Table S4). To further evaluate the effect of P-EBV on survival, we also tested different cut-offs used in prior studies for adult NPC using the ddPCR method, including 100, 200, and 1500 IU/mL, finding no correlation with EFS at any of these cut-offs (Supplemental Figure S1).

#### 3.2.2. Association of Pre-Treatment EBV DNA Levels with EFS and OS Among Patients Treated on GPOH

**Figure 4:**
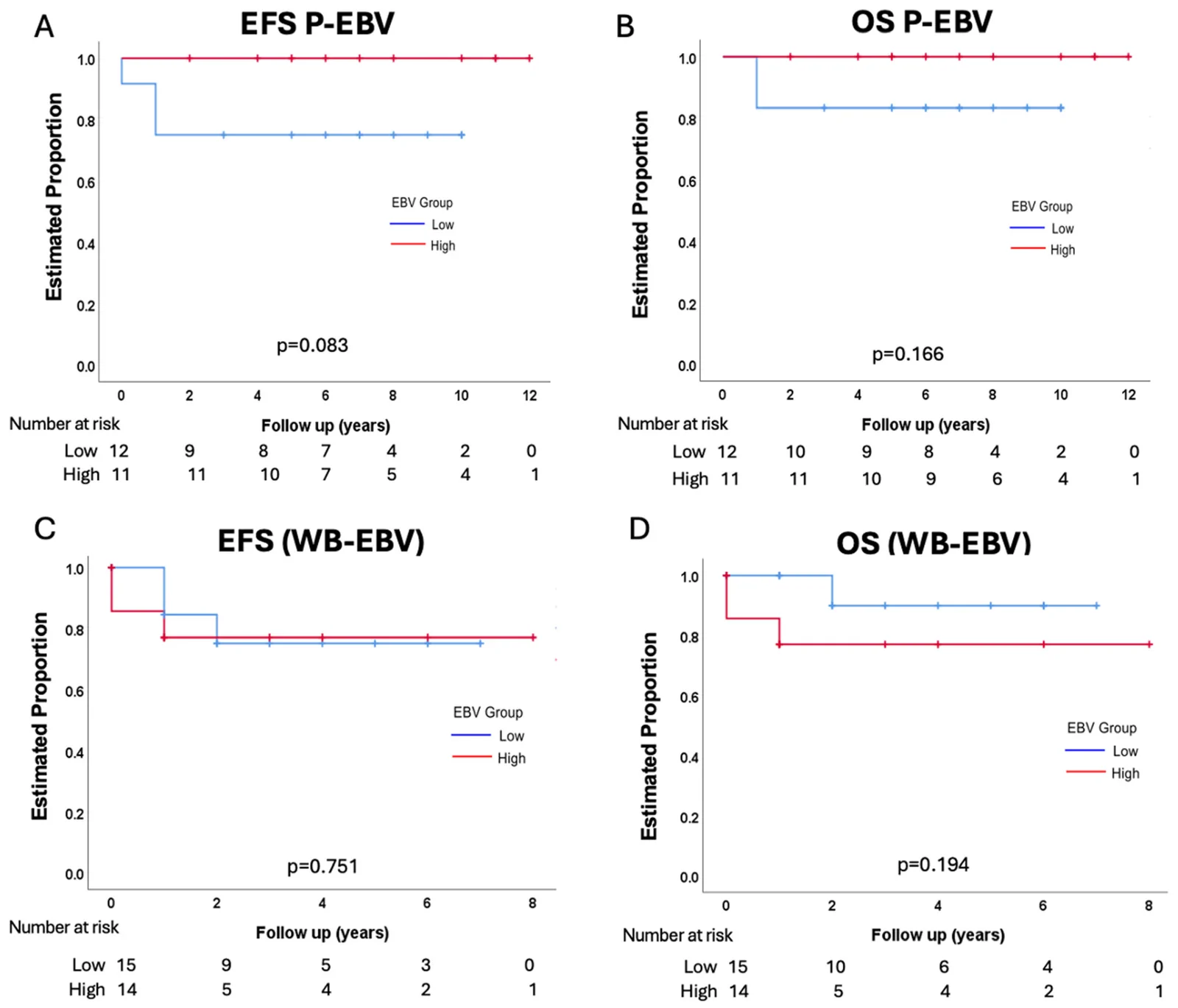
Survival analysis of patients treated by GPOH comparing high and low P-EBV DNA levels. (**A**) Kaplan–Meier curve showing the EFS of the studied NPC cases according to P-EBV. (**B**) Kaplan–Meier curve showing the OS of the studied NPC cases according to P-EBV. Total number of patients: 23, including 12 patients with low pre-treatment P-EBV and 11 patients with high pre-treatment P-EBV. (**C**) Kaplan–Meier curve showing the EFS of the studied NPC cases according to WB-EBV. (**D**) Kaplan–Meier curve showing the OS of the studied NPC cases according to WB-EBV. Total number of patients: 29, including 15 patients with low pre-treatment WB-EBV and 14 patients with high pre-treatment WB-EBV.

**Figure 5:**
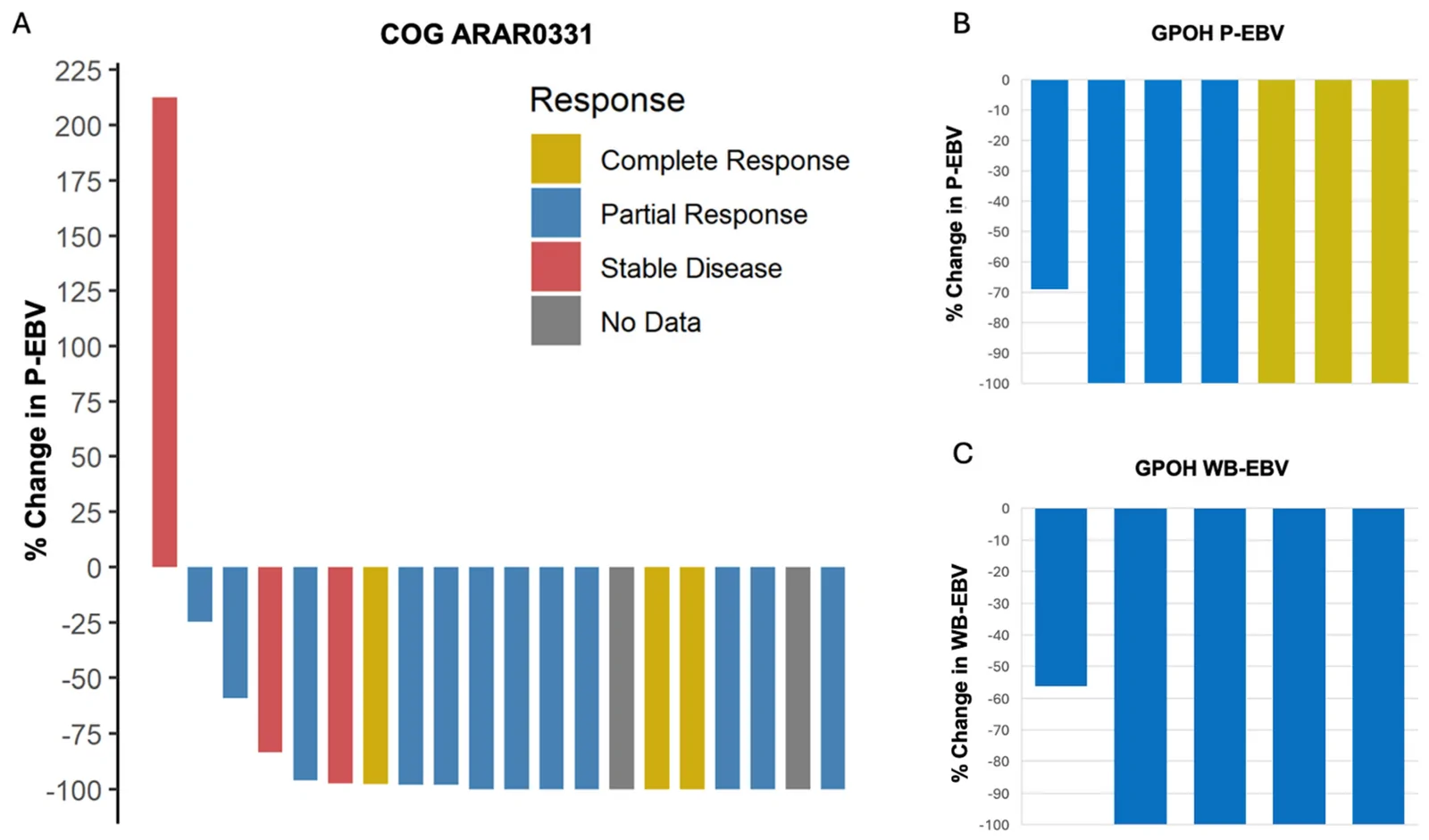
Waterfall plots of EBV DNA percent change post-induction chemotherapy for COG and GPOH cohorts using radiological response after induction chemotherapy. (**A**) Percent change from pre-treatment to post-induction and consolidation response for patients treated on COG study ARAR0331 (*n* = 20, Supplemental Table S6). (**B**) Percent change of EBV DNA post-induction chemotherapy in NPC patients with positive EBV DNA pre-treatment levels (GPOH-p-EBV cohort, *n* = 9, with 7 shown as 2 had negative pre-treatment and post-induction; Supplemental Table S8). (**C**) Percent change of EBV DNA post-induction chemotherapy in NPC patients with positive EBV DNA pre-treatment levels (GPOH-WB-EBV cohort, *n* = 13, with 5 shown as 8 had negative pre-treatment and post-induction; Supplemental Table S9).

Three patients in the P-EBV analysis cohort experienced events during follow-up, with all of them falling in the low group. There was no significant difference between patients with high and low P-EBV when evaluating 5-year EFS (Figure 4A) or 5-year OS (Figure 4B). Five-year EFS was 75.0 (+12.5)% for the low P-EBV group and 100.0 (+0.0)% for the high P-EBV group, with no significant difference between them (*p* = 0.083, Figure 4A). Five-year OS was 83.3 (+10.8)% for the low P-EBV group and 100.0 (+0.0)% for the high P-EBV group, with no significant difference between them (*p* = 0.166, Figure 4B). Cox regression analysis was not performed due to the small sample size.

In the WB-EBV cohort, six patients experienced events during follow-up (three in the low WB-EBV group, three in the high WB-EBV group). There was no significant difference between high and low WB-EBV when evaluating 5-year EFS (Figure 4C) or 5-year OS (Figure 4D). Five-year EFS was 74.0 (+13.2)% for the low WB-EBV group and 74.5 (+13.1)% for the high WB-EBV group (*p* = 0.751, Figure 4C). Five-year OS was 88.9 (+10.5)% for the low WB-EBV group and 74.0 (+13.2)% for the high WB-EBV group (*p* = 0.194, Figure 4D). Cox regression analysis was not performed due to the small sample size.

### 3.3. Percent Change of EBV DNA Levels After Induction Is Comparable Between COG and GPOH Cohorts

#### 3.3.1. Percent Change of P-EBV Comparing Pre-Treatment to Post-Induction Is Not Predictive of Response to Therapy for Patients on ARAR0331

For patients treated on ARAR0331, post-induction P-EBV DNA quantification was performed for 31 patients, and summary statistics for these patients can be found in Supplemental Table S5. In the P-EBV dataset from patients treated on ARAR0331, there were 20 patients with both pre-treatment and post-induction data available. A waterfall plot illustrating the percent change for each patient, along with their response after chemoradiotherapy, is provided in Figure 5A, with patient details for each of these cases in Supplemental Table S6. The median (range) percent change was −100 (−100 to 212.5). Of the 20 patients with matched pre-treatment and post-induction specimens, 18 were evaluable for response, while the remaining two came off protocol therapy early before response evaluation. Of the 18 evaluable patients, 15 responded (3 CR and 12 PR), and the remaining 3 had stable disease at the end of consolidation. While only 3 of the evaluable patients had stable disease, percent change does not qualitatively associate with response to therapy. Notably, the patient with a 212.5% increase had 24 IU/mL pre-induction and 75 IU/mL post-induction.

#### 3.3.2. Percent Change of P-EBV and WB-EBV DNA Levels Comparing Pre-Treatment and Post-Induction Did Not Correlate with Response for Patients Treated on GPOH

For patients treated on GPOH, there were 12 and 18 patients with post-induction P-EBV and WB-EBV DNA quantification performed, respectively, and summary statistics of these patients can be found in Supplemental Table S7. Pre-treatment and matched post-induction data were available for 9 patients from the P-EBV cohort (Supplemental Table S8). A waterfall plot illustrating the percent change of EBV DNA levels for each patient along with their response after induction chemotherapy is provided in Figure 5B, and response after chemoradiotherapy is also provided in Supplemental Figure S2A, with patient details for each of these cases in Supplemental Table S9. After induction chemotherapy, three patients achieved radiological CR, three very good PR (VGPR) and three PR. Seven patients had a positive pre-treatment level of P-EBV DNA, five of them turned to be negative after induction chemotherapy (3 were in CR, one in PR, and one in VGPR after induction chemotherapy; the latter suffered a relapse 9 months after the end of therapy with interferon), and the other two patients had a lower but persistent positive P-EBV DNA level (one was in VGPR and the other in PR after induction chemotherapy). Two patients had negative P-EBV both at the time of pre-treatment and post-induction, one in VGPR and the other in PR after induction chemotherapy. The median (range) percent change was −100 (−100.0 to −69.140).

In the WB-EBV cumulative dataset, there were 13 patients with both pre-treatment and post-induction data available (Supplemental Table S9). After induction chemotherapy, 1 patient achieved CR, 3 VGPR, and 9 PR. A waterfall plot illustrating the percent change of EBV DNA for each patient along with their response after induction chemotherapy is provided in Figure 5C, and response after chemoradiotherapy is also provided in Supplemental Figure S2B, with patient details for each of these cases in Supplemental Table S9. The median (range) percent change was −100.0 (−100.0 to −56.50). Five patients had pre-treatment positive levels of WB-EBV DNA, four of them turned to be negative after induction chemotherapy (all of them were in PR after induction chemotherapy), and the other patient had a lower but persistent positive WB-EBV DNA level and suffered a progressive disease and died 6 months after the end of radiotherapy. Eight patients had negative WB-EBV both at the time of pre-treatment and post-induction, and 1 was in CR, 3 in VGPR, and 4 in PR after induction chemotherapy. Of the 13 patients, 10 patients were in CR after the end of therapy (7 had persistent negative WB-EBV pre-treatment and post-induction, 1 died by suicide in CR, and 2 had positive pre-treatment and negative post-induction values). One patient had stable disease (positive pre-treatment EBV DNA value turned negative after induction chemotherapy), and one patient died after progression (had positive pre-treatment and post-induction chemotherapy EBV values).

After the end of induction chemotherapy, 3 of the 22 patients with available EBV levels from either P-EBV or WB-EBV cohorts still had positive EBV levels. One of these patients had progression after RT and died after having PR post-induction when their WB-EBV DNA level decreased from 1118 pre-treatment to 486 IU/mL. The other 2 patients are currently in CR despite continued positive P-EBV levels post-induction with 34 and 58 copies/mL.

## 4. Discussion

In this study, pre-treatment EBV DNA did not correlate with response or survival outcomes in children and adolescents with NPC treated in non-endemic regions. This study included quantitative values of EBV DNA from 102 patients pre-treatment and from 61 patients post-induction chemotherapy, making this analysis the largest for pediatric NPC patients from non-endemic areas [5,26]. Our study included newly diagnosed patients with NPC, comprising 50 patients from COG ARAR0331 representing North America, including United States and Canada (plus one patient from Australia), and 60 patients from GPOH, representing Germany and neighboring European countries. These patients had an age and gender distribution consistent with previous studies of childhood NPC [8,27,28]. Eleven patients within the GPOH group were older than 18 years at the time of diagnosis, but they received the pediatric treatment protocol and all had WHO type III histology of NPC, which is most commonly found in children and adolescents [2,3,27,28,29,30,31,32,33]. In the North American cohort from COG ARAR0331, there was a high percentage of black patients with pediatric NPC, as well as a higher incidence in males than females, with a ratio of 2.1:1. While GPOH studies did not have data for race or ethnicity, the male-to-female ratio was comparable at 2.2:1 in the NPC-2003 study (1.7:1 within the included patients of the GPOH study in this study). Both the North American and European cohorts are comparable to China, which has a reported male-to-female ratio of 2.5:1 [14,34].

Previous studies in adults have shown that EBV DNA levels in peripheral blood are significantly associated with NPC, and EBV viral load might reflect outcomes, including the risk of relapse and prognosis [30,35,36,37,38,39]. Our results show that 96% of pediatric NPC patients had a positive pre-treatment EBV DNA level within the ARAR0331 cohort and 65% in the GPOH cohorts. Previous studies have demonstrated a wide range of positive EBV DNA in patients with NPC, ranging from 34.3% to 97%. In most of these studies, EBV DNA was measured using plasma [27,40,41,42,43,44]. This wide range may be attributed to differences between endemic and non-endemic areas, different detection assays, different cut-offs for EBV DNA, clinical stages, patient age groups, and/or the histological subtype of the disease.

The results of our studies did not show a correlation between pre-treatment EBV DNA levels and age or stage of children with NPC, with the exception of higher EBV DNA levels in patients with stage IV within the GPOH WB-EBV DNA cohort. No relation was seen between pre-treatment EBV DNA and the presence of metastases at the time of diagnosis, which is contrary to prior studies of adults with NPC [45,46,47]. Our results also did not show a prognostic role for pre-treatment EBV DNA levels, which is contrary to findings from other studies in adults and children with NPC from endemic regions [6,7,12,33,36,39,44,48]. In both the COG and GPOH data, there was a trend indicating that low EBV DNA predicts worse outcomes, especially when using a cut off < 100 IU/mL in the COG data and <135 copies/mL in the GPOH P-EBV cohort. This contrasts with what is seen in adult NPC.

We evaluated the percent change of EBV levels and their association with outcomes and found no significant difference, though this analysis is limited by the number of patients with events, including progressive disease or death from NPC. Importantly, other studies have demonstrated that the post-treatment level of EBV DNA is a valuable prognostic marker that correlates with residual disease, prediction of relapse, and increased risk for tumor mortality [26,47,48,49]. This difference may be related to the different time points, as in our study we analyzed the values of EBV DNA post-induction chemotherapy and not after the end of the whole therapy (chemoradiotherapy and/or maintenance). In pediatric NPC, a retrospective study found that a high P-EBV DNA load pre-treatment serves as an independent prognostic factor, correlating with poorer clinical outcomes in patients less than 22 years of age [12]. This study only included patients from endemic areas in China with more high-grade histological types, advanced staging classifications (86.5% T3–T4 in their cohort), and aggressive clinical behavior, with patients treated with a median radiation dose of 70 Gy. In contrast, our study reveals that among the lower prevalence of high-grade NPC in our population, P-EBV does not offer predictive power regarding patient prognosis. This disparity suggests that the predictive significance of P-EBV may be contingent upon specific tumor characteristics that vary by region and grade. As the GPOH protocol, in contrast to the COG study, includes maintenance immunotherapy with interferon for 6 months after completion of chemoradiotherapy, the value of EBV DNA after the end of chemoradiotherapy and end of maintenance therapy must be analyzed in further detail.

Incorporating pre-treatment P-EBV DNA levels significantly improved prognostic prediction in adult NPC [9]. However, P-EBV has never been officially added to the AJCC/UICC Staging Systems. WHO guidelines for NPC include the use of EBV to prognosticate response both at diagnosis and after treatment, and EBV DNA levels can be used to determine therapy or assess early progression after treatment [50,51]. However, a major limitation of using EBV DNA for prognosis and treatment planning is the absence of a standardized, reproducible method for EBV DNA quantification across laboratories [11]. Both plasma and whole blood have been used for circulating EBV DNA quantification; however, measurements obtained from plasma or whole blood are not directly interchangeable because they represent overlapping but biologically distinct viral DNA compartments. Whole blood contains both cell-free and cell-associated EBV DNA and may provide greater detection sensitivity, whereas plasma predominantly contains cell-free EBV DNA and may more specifically reflect tumor-derived DNA [52]. In addition to different sample types (whole blood vs. plasma), nearly every component of the methods used for EBV measurement in this study differed between cohorts, including input sample volume, extraction method, sequence target (single copy vs. multi-copy), master mix, amplification technique (qPCR vs. ddPCR), and the material used for calibration. Because of these methodological differences, the results from these cohorts cannot be directly compared, posing substantial challenges for data harmonization and interpretation.

We also found that post-induction EBV DNA was not qualitatively associated with response, which may indicate that post-induction EBV DNA levels might be ineffective for stratifying patients under 19 years of age for treatment, especially radiation. This requires further study with a larger cohort so that statistical analyses of sufficient power can be performed. An important limitation is the methodological heterogeneity in EBV DNA measurement, including differences in sample matrix and analytical platform. Although the analyses were conducted separately using assay-specific cutoffs and no cross-cohort quantitative comparison was performed, methodological differences may still have contributed to variation in effect estimates. Therefore, the reported cutoffs should be considered specific to their respective analytical workflows and should not be transferred to other specimen types, platforms, or laboratories without validation. Prospective studies using standardized specimen processing, harmonized assays, and common reference materials are needed to establish broadly applicable clinical thresholds. Other limitations of this study were mainly related to its retrospective nature, deficiency of some comparative data points (race and ethnicity within the GPOH group), and the small sample size compared to studies done in endemic areas due to the rarity of NPC in North America and Europe. This study included patients diagnosed as early as 2003, as prior to that time, EBV-PCR was not routinely done at the time of diagnosis or during follow-up in many centers. Only 200 μL of plasma was used for both COG and GPOH P-EBV samples, while others report best results with >600 μL tested. Detection of EBV DNA from GPOH patients was done from different samples (plasma or whole blood) in different laboratories (Luebeck, Giessen, or Hannover). Since the linear range of the method within the WB-EBV samples in the GPOH cohort starts at 3200 IU/mL, numerical values below 3200 IU/mL lie outside the evaluable range and cannot be evaluated quantitatively, except for the calculation of the median. Also, due to the different sensitivity cutoff levels for detecting EBV DNA within the different samples and measurements (plasma vs. whole blood), we considered negative EBV-PCR results with no available exact value to be zero IU/mL for this analysis. Despite these limitations, this study provides a valuable reference for NPC in children in non-endemic regions. However, prospective studies are required to validate our findings.

## 5. Conclusions

In this study, we address an urgent need to evaluate the prognostic value of EBV DNA levels in pediatric NPC among patients in non-endemic regions, including North America and Europe. EBV DNA is predictive of treatment response and outcomes in adult NPC in both endemic and non-endemic regions, as well as in pediatric NPC from endemic regions. The COG and GPOH collaborated to retrospectively evaluate the ability of plasma and whole blood EBV DNA to predict treatment response and outcomes in pediatric NPC.

We did not detect a statistically significant association between EBV DNA and clinical outcomes in pediatric NPC patients from non-endemic regions, including the United States, Canada, Germany, Austria, Switzerland and the Netherlands. Although EBV DNA is incorporated into WHO guidelines for prognostication in adults with NPC, our findings suggest that using EBV DNA levels to predict treatment response or outcomes in children and adolescents in non-endemic regions may not be useful. These results also highlight the need for further studies to clarify the role of EBV DNA in children and adolescents with NPC outside endemic regions and to better understand how non-endemic pediatric NPC may differ from adult NPC and from pediatric NPC in endemic settings.

## Figures and Tables

**Table 1:** Patient characteristics of all cohorts included in the EBV DNA analysis performed, including the Children’s Oncology Group (COG) ARAR0331 and the German Society of Pediatric Oncology and Hematology (GPOH).

Characteristics	North America	Europe (GPOH Cohorts)		
	COG ARAR0331 (*n* = 50)	Overall (N = 60)	P-EBV (N = 26)	WB-EBV (N = 34)
Age at enrollment (years) mean	13	15	13	15
Median (range)	14 (3–18)	15.1 (9–25)	14.59 (9–25)	15.78 (9–24)
Sex, *n* (%)				
Female	17 (34)	22	8 (31)	14 (41)
Male	33 (66)	38	18 (69)	20 (59)
Race, *n* (%)				
Black	22 (44)	0 (0)	0 (0)	0 (0)
White	22 (44)	0 (0)	0 (0)	0 (0)
Unknown	6 (12)	60 (100)	26 (100)	34 (100)
Stage of disease, *n* (%)				
I	0	0	0	0
II	4 (8)	3 (5)	1 (4)	2 (6)
III	19 (38)	8 (13)	1 (4)	7 (21)
IV	27 (54) *	49 (82)	24 (92)	25 (73)
GPOH NPC-Study				
NPC-2003	NA	22	22	0
Interim	NA	21	4	17
NPC-2016	NA	17	0	17

* Stage IV includes IVA (*n* = 12), IVB (*n* = 10), and IVC (*n* = 5). NA indicates not applicable.

## Data Availability

The COG ARAR0331 data has been registered in dbGaP (phs003092.v1.p1) and all data will be available at dbGaP. The GPOH data are not readily available due to patient privacy concerns. Requests to access the datasets should be directed to Dr. Udo Kontny.

## Supplementary Materials

The following supporting information can be downloaded at https://www.mdpi.com/article/10.3390/cancers18172800/s1: Tables S1–S9 and Figures S1 and S2.

## Abbreviations

The following abbreviations are used in this manuscript:

NPC	nasopharyngeal carcinoma
EBV	Epstein–Barr virus
COG	Children’s Oncology Group
GPOH	German Society of Pediatric Oncology and Hematology
P-EBV	plasma EBV DNA
WB-EBV	whole-blood EBV DNA
OS	overall survival
DFS	disease-free survival
EFS	event free survival
RECIST	Response Evaluation Criteria in Solid Tumors
IFNβ	Interferon-Beta
IRB	Institutional Review Board
RWTH	Rheinisch-Westfälische Technische Hochschule
AJCC	American Joint Cancer Classification
MRI	magnetic resonance imaging
WHO	World Health Organization
ddPCR	droplet digital PCR
RQ-PCR	real-time quantitative polymerase chain reaction
ATCC	American Type Culture Collection
CR	complete response
PR	partial response
VGPR	very good partial response

## References

[1] Su Z.Y., Siak P.Y., Lwin Y.Y., Cheah S.-C. (2024). Epidemiology of nasopharyngeal carcinoma: Current insights and future outlook. Cancer Metastasis Rev..

[2] Kontny U., Franzen S., Behrends U., Bührlen M., Christiansen H., Delecluse H., Eble M., Feuchtinger T., Gademann G., Granzen B. (2016). Diagnosis and Treatment of Nasopharyngeal Carcinoma in Children and Adolescents—Recommendations of the GPOH-NPC Study Group. Klin. Padiatr..

[3] Sultan I., Casanova M., Ferrari A., Rihani R., Rodriguez-Galindo C. (2010). Differential features of nasopharyngeal carcinoma in children and adults: A SEER study. Pediatr. Blood Cancer.

[4] Wu B., Shen L., Peng G., Li Y., Zhou Z., Li J., Huang X., Zhou Q., Jiang H., Huang J. (2022). Molecular characteristics of pediatric nasopharyngeal carcinoma using whole-exome sequencing. Oral Oncol..

[5] Matalka I., Al Hamad M., Al-Hussaini M., Alzoubi F.Q. (2012). The incidence of Epstein-Barr virus in nasopharyngeal carcinoma of Jordanian patients. Eur. Arch. Otorhinolaryngol..

[6] Chua M.L.K., Wee J.T.S., Hui E.P., Chan A.T.C. (2016). Nasopharyngeal carcinoma. Lancet.

[7] Liu S.L., Sun X.S., Li X.Y., Tang L.Q., Chen Q.Y., Lin H.X., Liang Y.J., Yan J.J., Lin C., Guo S.S. (2019). The diagnostic and prognostic values of plasma Epstein-Barr virus DNA for residual cervical lymphadenopathy in nasopharyngeal carcinoma patients: A retrospective study. Cancer Commun..

[8] Jouin A., Helfre S., Bolle S., Claude L., Laprie A., Bogart E., Vigneron C., Potet H., Ducassou A., Claren A. (2019). Adapted strategy to tumor response in childhood nasopharyngeal carcinoma: The French experience. Strahlenther. Onkol..

[9] Lee V.H., Kwong D.L., Leung T.W., Choi C.W., O’Sullivan B., Lam K.O., Lai V., Khong P.L., Chan S.K., Ng C.Y. (2019). The addition of pretreatment plasma Epstein-Barr virus DNA into the eighth edition of nasopharyngeal cancer TNM stage classification. Int. J. Cancer.

[10] Xu C., Chen Y.P., Liu X., Li W.F., Chen L., Mao Y.P., Zhang Y., Guo R., Zhou G.Q., Tang L.L. (2017). Establishing and applying nomograms based on the 8th edition of the UICC/AJCC staging system to select patients with nasopharyngeal carcinoma who benefit from induction chemotherapy plus concurrent chemoradiotherapy. Oral Oncol..

[11] Lee A.W.M., Lee V.H.F., Ng W.-T., Strojan P., Saba N.F., Rinaldo A., Willems S.M., Rodrigo J.P., Forastiere A.A., Ferlito A. (2021). A systematic review and recommendations on the use of plasma EBV DNA for nasopharyngeal carcinoma. Eur. J. Cancer.

[12] Shen T., Tang L.Q., Gu W.G., Luo D.H., Chen Q.Y., Li P.J., Mai D.M., Mai H.Q., Mo H.Y. (2015). Plasma Epstein-Barr Viral Deoxyribonucleic Acid Predicts Worse Outcomes in Pediatric Nonmetastatic Nasopharyngeal Carcinoma Patients: An Observational Study of 89 Cases in an Endemic Area. Medicine.

[13] Dholaria H., Tsetlina V., Simpson S.K., Gillies E., Eswaran N., Rodriguez-Galindo C., Schultz K.A.P., Chen K.S., Wu C.C., Krasin M.J. (2025). One-Two Punch: Combining Chemotherapy and Immunotherapy to Decrease Radiation Dose and Related Toxicity in Children and Adolescents with Nasopharyngeal Carcinoma. Clin. Cancer Res..

[14] Carlos R.-G., Krailo M.D., Krasin M.J., Huang L., Beth McCarville M., Hicks J., Pashankar F., Pappo A.S. (2019). Treatment of Childhood Nasopharyngeal Carcinoma With Induction Chemotherapy and Concurrent Chemoradiotherapy: Results of the Children’s Oncology Group ARAR0331 Study. J. Clin. Oncol..

[15] Buehrlen M., Zwaan C.M., Granzen B., Lassay L., Deutz P., Vorwerk P., Staatz G., Gademann G., Christiansen H., Oldenburger F. (2012). Multimodal treatment, including interferon beta, of nasopharyngeal carcinoma in children and young adults: Preliminary results from the prospective, multicenter study NPC-2003-GPOH/DCOG. Cancer.

[16] Römer T., Franzen S., Kravets H., Farrag A., Makowska A., Christiansen H., Eble M.J., Timmermann B., Staatz G., Mottaghy F.M. (2022). Multimodal Treatment of Nasopharyngeal Carcinoma in Children, Adolescents and Young Adults-Extended Follow-Up of the NPC-2003-GPOH Study Cohort and Patients of the Interim Cohort. Cancers.

[17] Römer T., Vokuhl C., Staatz G., Mottaghy F.M., Christiansen H., Eble M.J., Timmermann B., Klussmann J.P., Elbracht M., Calaminus G. (2024). Combination of nivolumab with standard induction chemotherapy in children and adults with EBV-positive nasopharyngeal carcinoma: Protocol of a prospective multicenter phase 2 trial. HNO.

[18] Zhang W., Chen Y., Chen L., Guo R., Zhou G., Tang L., Mao Y., Li W., Liu X., Du X. (2015). The clinical utility of plasma Epstein-Barr virus DNA assays in nasopharyngeal carcinoma: The dawn of a new era?: A systematic review and meta-analysis of 7836 cases. Medicine.

[19] Liu T.B., Zheng Z.H., Pan J., Pan L.L., Chen L.H. (2017). Prognostic role of plasma Epstein-Barr virus DNA load for nasopharyngeal carcinoma: A meta-analysis. Clin. Investig. Med..

[20] Taverna F., Alfieri S., Romano R., Campanini G., Marceglia S., Giardina F., Mazzocchi A., Comoli P., Gloghini A., Quattrone P. (2022). Comparing BamHI-W and CE-marked assays to detect circulating Epstein-Barr Virus (EBV) DNA of nasopharyngeal cancer patients in a non-endemic area. Oral Oncol..

[21] Miller J.A., Huang C., Yamamoto F., Sahoo M.K., Le Q.T., Pinsky B.A. (2023). Comparison of Real-Time PCR and Digital PCR for Detection of Plasma Epstein-Barr Virus DNA in Nasopharyngeal Carcinoma. J. Mol. Diagn..

[22] Trypsteen W., Vynck M., De Neve J., Bonczkowski P., Kiselinova M., Malatinkova E., Vervisch K., Thas O., Vandekerckhove L., De Spiegelaere W. (2015). ddpcRquant: Threshold determination for single channel droplet digital PCR experiments. Anal. Bioanal. Chem..

[23] Wagner H.J., Wessel M., Jabs W., Smets F., Fischer L., Offner G., Bucsky P. (2001). Patients at risk for development of posttransplant lymphoproliferative disorder: Plasma versus peripheral blood mononuclear cells as material for quantification of Epstein-Barr viral load by using real-time quantitative polymerase chain reaction. Transplantation.

[24] Heid C.A., Stevens J., Livak K.J., Williams P.M. (1996). Real time quantitative PCR. Genome Res..

[25] Engelmann I., Petzold D.R., Kosinska A., Hepkema B.G., Schulz T.F., Heim A. (2008). Rapid quantitative PCR assays for the simultaneous detection of herpes simplex virus, varicella zoster virus, cytomegalovirus, Epstein-Barr virus, and human herpesvirus 6 DNA in blood and other clinical specimens. J. Med. Virol..

[26] Stoker S.D., Wildeman M.A., Novalic Z., Fles R., van der Noort V., de Bree R., Braunius W.W., van den Broek G.B., Kreike B., Kross K.W. (2016). Can Epstein-Barr virus DNA load in nasopharyngeal brushings or whole blood predict recurrent nasopharyngeal carcinoma in a non-endemic region? A prospective nationwide study of the Dutch Head and Neck Oncology Cooperative Group. Eur. Arch. Otorhinolaryngol..

[27] Zhang J., Luo X., Huang Q., Huang Y. (2021). Clinicopathological and prognostic features of nasopharyngeal carcinoma in children and adolescents: A retrospective study of 196 cases in South China. Int. J. Cancer.

[28] Zekri W., Wahed M.A., Attia E., Khalil E. (2020). Pediatric Nasopharyngeal Carcinoma: A Rare Tumor in a Developing Country-What Do We Learn?. J. Pediatr. Hematol. Oncol..

[29] Yan Z., Xia L., Huang Y., Chen P., Jiang L., Zhang B. (2013). Nasopharyngeal carcinoma in children and adolescents in an endemic area: A report of 185 cases. Int. J. Pediatr. Otorhinolaryngol..

[30] Yip T.T., Ngan R.K., Fong A.H., Law S.C. (2014). Application of circulating plasma/serum EBV DNA in the clinical management of nasopharyngeal carcinoma. Oral Oncol..

[31] Richards M.K., Dahl J.P., Gow K., Goldin A.B., Doski J., Goldfarb M., Nuchtern J., Langer M., Beierle E.A., Vasudevan S. (2016). Factors Associated With Mortality in Pediatric vs Adult Nasopharyngeal Carcinoma. JAMA Otolaryngol. Head Neck Surg..

[32] Zeng Z., Chen C., Guo L., Zhang C., Chen L., Yuan C., Lu L. (2020). Exploring the Optimal Chemotherapy Strategy for Locoregionally Advanced Children and Adolescent Nasopharyngeal Carcinoma Based on Pretreatment Epstein-Barr Virus DNA Level in the Era of Intensity Modulated Radiotherapy. Front. Oncol..

[33] Qiu W., Lv X., Guo X., Yuan Y. (2020). Clinical Implications of Plasma Epstein-Barr Virus DNA in Children and Adolescent Nasopharyngeal Carcinoma Patients Receiving Intensity-Modulated Radiotherapy. Front. Oncol..

[34] Chen W., Zheng R., Baade P.D., Zhang S., Zeng H., Bray F., Jemal A., Yu X.Q., He J. (2016). Cancer statistics in China, 2015. CA Cancer J. Clin..

[35] Chan K.C.A., Woo J.K.S., King A., Zee B.C.Y., Lam W.K.J., Chan S.L., Chu S.W.I., Mak C., Tse I.O.L., Leung S.Y.M. (2017). Analysis of Plasma Epstein-Barr Virus DNA to Screen for Nasopharyngeal Cancer. N. Engl. J. Med..

[36] Leung S.F., Zee B., Ma B.B., Hui E.P., Mo F., Lai M., Chan K.C., Chan L.Y., Kwan W.H., Lo Y.M. (2006). Plasma Epstein-Barr viral deoxyribonucleic acid quantitation complements tumor-node-metastasis staging prognostication in nasopharyngeal carcinoma. J. Clin. Oncol..

[37] Li W.F., Zhang Y., Huang X.B., Du X.J., Tang L.L., Chen L., Peng H., Guo R., Sun Y., Ma J. (2017). Prognostic value of plasma Epstein-Barr virus DNA level during posttreatment follow-up in the patients with nasopharyngeal carcinoma having undergone intensity-modulated radiotherapy. Chin. J. Cancer.

[38] Savitri E., Haryana M.S. (2015). Expression of interleukin-8, interleukin-10 and Epstein-Barr viral-load as prognostic indicator in nasopharyngeal carcinoma. Glob. J. Health Sci..

[39] Zeng M.C., Jia Q.J., Xu J., Chen J.J., Qin K., Xu L.M. (2023). The whole-blood Epstein-Barr virus DNA can serve as a valuable molecular marker for diagnosis and prognosis prediction of nasopharyngeal carcinoma. Am. J. Cancer Res..

[40] Liang S.B., Zhang N., Chen D.M., Yang X.L., Chen B.H., Zhao H., Lu R.L., Chen Y., Fu L.W. (2019). Prognostic value of gross tumor regression and plasma Epstein Barr Virus DNA levels at the end of intensity-modulated radiation therapy in patients with nasopharyngeal carcinoma. Radiother. Oncol..

[41] Nicholls J.M., Lee V.H., Chan S.K., Tsang K.C., Choi C.W., Kwong D.L., Lam K.O., Chan S.Y., Tong C.C., So T.H. (2019). Negative plasma Epstein-Barr virus DNA nasopharyngeal carcinoma in an endemic region and its influence on liquid biopsy screening programmes. Br. J. Cancer.

[42] Tan L.P., Tan G.W., Sivanesan V.M., Goh S.L., Ng X.J., Lim C.S., Kim W.R., Mohidin T., Mohd Dali N.S., Ong S.H. (2020). Systematic comparison of plasma EBV DNA, anti-EBV antibodies and miRNA levels for early detection and prognosis of nasopharyngeal carcinoma. Int. J. Cancer.

[43] Fung S.Y., Lam J.W., Chan K.C. (2016). Clinical utility of circulating Epstein-Barr virus DNA analysis for the management of nasopharyngeal carcinoma. Chin. Clin. Oncol..

[44] Peng L., Yang Y., Guo R., Mao Y.P., Xu C., Chen Y.P., Sun Y., Ma J., Tang L.L. (2018). Relationship between pretreatment concentration of plasma Epstein-Barr virus DNA and tumor burden in nasopharyngeal carcinoma: An updated interpretation. Cancer Med..

[45] Gurtsevitch V.E., Senyuta N.B., Ignatova A.V., Lomaya M.V., Kondratova V.N., Pavlovskaya A.I., Dushenkina T.E., Maximovich D.M., Smirnova K.V., Mudunov A.M. (2017). Epstein-Barr virus biomarkers for nasopharyngeal carcinoma in non-endemic regions. J. Gen. Virol..

[46] Gihbid A., Benzeid R., Faouzi A., Nourlil J., Tawfiq N., Benchakroun N., Guensi A., Bendahhou K., Benider A., El Benna N. (2021). Circulating cell-free epstein-barr virus DNA levels and clinical features in Moroccan patients with nasopharyngeal carcinoma. Infect. Agents Cancer.

[47] Lin J.C., Wang W.Y., Chen K.Y., Wei Y.H., Liang W.M., Jan J.S., Jiang R.S. (2004). Quantification of plasma Epstein-Barr virus DNA in patients with advanced nasopharyngeal carcinoma. N. Engl. J. Med..

[48] Qu H., Huang Y., Zhao S., Zhou Y., Lv W. (2020). Prognostic value of Epstein-Barr virus DNA level for nasopharyngeal carcinoma: A meta-analysis of 8128 cases. Eur. Arch. Otorhinolaryngol..

[49] Lv J.W., Zhou G.Q., Li J.X., Tang L.L., Mao Y.P., Lin A.H., Ma J., Sun Y. (2017). Magnetic Resonance Imaging-Detected Tumor Residue after Intensity-Modulated Radiation Therapy and its Association with Post-Radiation Plasma Epstein-Barr Virus Deoxyribonucleic Acid in Nasopharyngeal Carcinoma. J. Cancer.

[50] Wang W.Y., Twu C.W., Chen H.H., Jan J.S., Jiang R.S., Chao J.Y., Liang K.L., Chen K.W., Wu C.T., Lin J.C. (2010). Plasma EBV DNA clearance rate as a novel prognostic marker for metastatic/recurrent nasopharyngeal carcinoma. Clin. Cancer Res..

[51] Li W.Z., Wu H.J., Lv S.H., Hu X.F., Liang H., Liu G.Y., Lu N., Bei W.X., Lv X., Guo X. (2021). Assessment of Survival Model Performance Following Inclusion of Epstein-Barr Virus DNA Status in Conventional TNM Staging Groups in Epstein-Barr Virus-Related Nasopharyngeal Carcinoma. JAMA Netw. Open.

[52] Rzepka M., Depka D., Gospodarek-Komkowska E., Bogiel T. (2023). Diagnostic Value of Whole-Blood and Plasma Samples in Epstein-Barr Virus Infections. Diagnostics.

