# Supplementary material for: Evaluating the Prognostic Relevance of Pre-Treatment Epstein–Barr Virus Levels in Non-Endemic Pediatric Nasopharyngeal Carcinoma
Source: Cancers (Basel). 2026 Aug 28;18(17):2800. doi: 10.3390/cancers18172800 (PMC13565518; doi:10.3390/cancers18172800)
Supplement: Supplementary file 1 [file cancers-18-02800-s001.zip › cancers-4451964-supplementary.pdf]

Supplemental Material

Supplemental Table S1: Patient characteristics of 50 pre-treatment P-EBV patients from COG ARAR0331 by group dichotomized by high and low P-EBV using median (1,218.5 IU/ml) as the cut-off.

Characteristics	Low (N=25) N (%)	High (N=25) N(%)	P value
Age at Enrollment (Years			0.4592[1]
Mean	13	14	
Median (Range)	14 (3, 17)	14 (8, 18)	
Sex			1.0000[2]
Female	8 (32.0%)	9 (36.0%)	
Male	17 (68.0%)	16 (64.0%)	
Race			0.5724[2]
Black	13 (52.0%)	9 (36.0%)	
White	9 (36.0%)	13 (52.0%)	
Other/Unknown	3 (12.0%)	3 (12.0%)	
Ethnicity			1.0000[2]
Hispanic or Latino	1 (4.0%)	2 (8.0%)	
Not Hispanic or Latino	24 (96.0%)	23 (92.0%)	
AJCC Stage			0.5463[2]
Stage IIB	3 (12.0%)	1 (4.0%)	
Stage III	7 (28.0%)	12 (48.0%)	
Stage IVA	7 (28.0%)	5 (20.0%)	
Stage IVB	6 (24.0%)	4 (16.0%)	
Stage IVC	2 (8.0%)	3 (12.0%)	

Supplemental Table S2: Patient characteristics of pre-treatment P-EBV GPOH cohort using median as cut off (n=23) Quantitative data are presented as mean ± SD and median (range), qualitative data are presented as number (percentage), Significance defined by $p < 0.05$. Mann Whitney U test was used for comparing continuous data. Chi square ($\chi 2$) test and Fisher Exact test were used for comparing categorical data. GPOH indicates German Pediatric Oncology Hematology group, EBV Epstein Barr virus, WB-EBV whole blood Epstein Barr virus, T Tumor stage, N Lymph node stage, and M metastasis.

GPOH Characteristics		P-EBV						
		Total, n=23		Low P-EBV (≤135 copies/ml), n=12		High P-EBV (>135 copies/ml), n=11		P value
Age (years)	Mean ± SD	14.63 ± 3.05		14.63 ± 2.17		14.63 ± 3.91		0.566 [M]
	Median (range)	13.6 (10.8 – 25.2)		14.8 (11.6 – 18.8)		13.4 (10.8 – 25.2)		
Age groups	<15 years	15	(65.2%)	6	(50.0%)	9	(81.8%)	0.193 [F]
	≥15 years	8	(34.8%)	6	(50.0%)	2	(18.2%)	
Sex	Male	17	(73.9%)	10	(83.3%)	7	(63.6%)	0.371 [F]
	Female	6	(26.1%)	2	(16.7%)	4	(36.4%)	
Stage of disease	Stage II	1	(4.3%)	1	(8.3%)	0	(0.0%)	0.999 [F]
	Stage III	1	(4.3%)	1	(8.3%)	0	(0.0%)	
	Stage IV	21	(91.3%)	10	(83.3%)	11	(100.0%)	
Stage of disease combined	Stage II + Stage III	2	(8.7%)	2	(16.7%)	0	(0.0%)	0.478 [F]
	Stage IV	21	(91.3%)	10	(83.3%)	11	(100.0%)	
T	T1	4	(17.4%)	3	(25.0%)	1	(9.1%)	0.517 [F]
	T2	2	(8.7%)	0	(0.0%)	2	(18.2%)	
	T3	8	(34.8%)	4	(33.3%)	4	(36.4%)	
	T4	9	(39.1%)	5	(41.7%)	4	(36.4%)	
T combined	T1/T2/T3	14	(60.9%)	7	(58.3%)	7	(63.6%)	0.999 [F]
	T4	9	(39.1%)	5	(41.7%)	4	(36.4%)	
N	0	1	(4.3%)	1	(8.3%)	0	(0.0%)	0.478 [F]
	1	2	(8.7%)	2	(16.7%)	0	(0.0%)	
	2	19	(82.6%)	9	(75.0%)	10	(90.9%)	
	3	1	(4.3%)	0	(0.0%)	1	(9.1%)	
N combined	N0/N1/N2	22	(95.7%)	12	(100.0%)	10	(90.9%)	0.478 [F]
	N3	1	(4.3%)	0	(0.0%)	1	(9.1%)	
M	M0	22	(95.7%)	12	(100.0%)	10	(90.9%)	0.478 [F]
	M1	1	(4.3%)	0	(0.0%)	1	(9.1%)	

Supplemental Table S3: Patient characteristics of pre-treatment whole blood-EBV (WB-EBV) GPOH cohort using median as cut off (n=29). Quantitative data are presented as mean ± SD and median (range), qualitative data are presented as number (percentage), Significance defined by $p < 0.05$. Student t test was used for comparing continuous data. Chi square ($\chi 2$) test and Fisher Exact test were used for comparing categorical data.

GPOH Characteristics		WB-EBV						
		Total, n=29		Low WB-EBV (≤53 IU/ml), n=15		High WB-EBV (>53 IU/ml), n=14		P value
Age (years)	Mean ± SD	16.22 ± 3.13		16.88 ± 2.99		15.52 ± 3.23		0.247 [t]
	Median (range)	16 (11.2 – 24.9)		17 (12.3 – 24.9)		15.5 (11.2 – 22.8)		
Age groups	<15 years	10	(34.5%)	4	(26.7%)	6	(42.9%)	0.450 [F]
	≥15 years	19	(65.5%)	11	(73.3%)	8	(57.1%)	
Sex	Male	16	(55.2%)	9	(60.0%)	7	(50.0%)	0.588 [χ2]
	Female	13	(44.8%)	6	(40.0%)	7	(50.0%)	
Stage of disease	Stage II	2	(6.9%)	2	(13.3%)	0	(0.0%)	0.017 [F]
	Stage III	7	(24.1%)	6	(40.0%)	1	(7.1%)	
	Stage IV	20	(69.0%)	7	(46.7%)	13	(92.9%)	
Stage of disease combined	Stage II + Stage III	9	(31.0%)	8	(53.3%)	1	(7.1%)	0.014 [F]
	Stage IV	20	(69.0%)	7	(46.7%)	13	(92.9%)	
T	T1	2	(7.4%)	2	(14.3%)	0	(0.0%)	0.095 [F]
	T2	3	(11.1%)	3	(21.4%)	0	(0.0%)	
	T3	12	(44.4%)	6	(42.9%)	6	(46.2%)	
	T4	10	(37.0%)	3	(21.4%)	7	(53.8%)	
T combined	T1/T2/T3	17	(63.0%)	11	(78.6%)	6	(46.2%)	0.120 [F]
	T4	10	(37.0%)	3	(21.4%)	7	(53.8%)	
N	0	1	(3.6%)	1	(6.7%)	0	(0.0%)	0.571 [F]
	1	3	(10.7%)	2	(13.3%)	1	(7.7%)	
	2	20	(71.4%)	11	(73.3%)	9	(69.2%)	
	3	4	(14.3%)	1	(6.7%)	3	(23.1%)	
N combined	N0/N1/N2	24	(85.7%)	14	(93.3%)	10	(76.9%)	0.311 [F]
	N3	4	(14.3%)	1	(6.7%)	3	(23.1%)	
M	M0	23	(79.3%)	14	(93.3%)	9	(64.3%)	0.080 [F]
	M1	6	(20.7%)	1	(6.7%)	5	(35.7%)	

Supplemental Figure S1: Survival Analysis from 50 patients treated on ARAR0331 comparing different cutoffs (A) 100 (B) 200, and (C) 1500 IU/mL for P-EBV DNA levels using ddPCR.

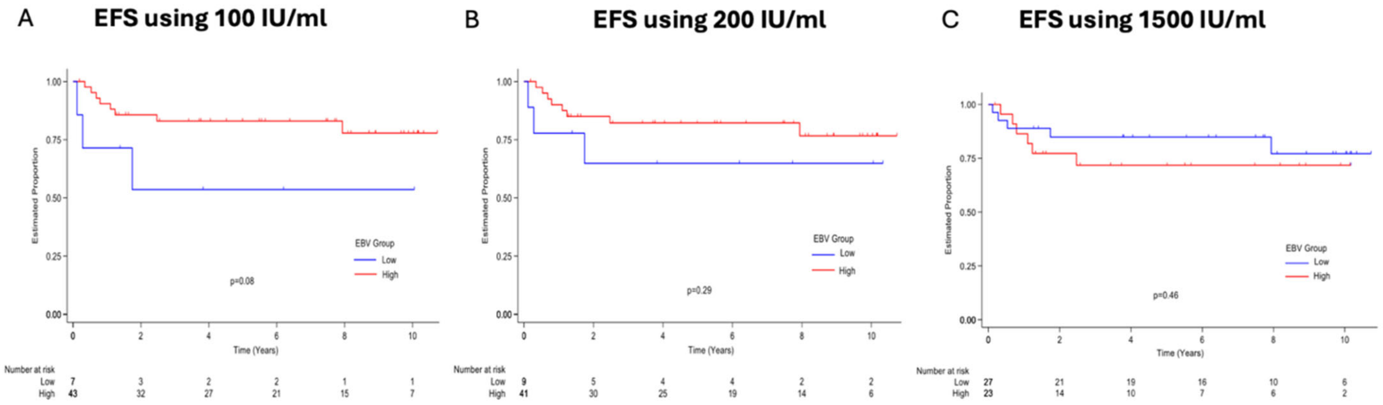

Supplemental Table S4 - Multivariate Cox Analysis of pre-treatment P-EBV from ARAR0331 associated with EFS, adjusted for age, race, and grouped stage. (n=50, HR = 1.12, 95% CI: 0.32–3.97; p = 0.86).

Characteristics	HR (95% CI)	P-Value
EBV group		0.86
Low	Reference	
High	1.12 (0.32 - 3.97)	
Age at Enrollment (Years)	0.82 (0.68 - 0.99)	0.04
Race		0.01
Black	Reference	
White	1.44 (0.31 - 6.75)	
Other/Unknown	10.4 (2.09 – 51.8)	
Stage group		0.86
Stages IIb-III	Reference	
Stage IV	1.13 (0.28 - 4.51)	

Supplemental Table S5: Patient characteristics of 31 patients treated on ARAR0331 with post- induction P-EBV DNA quantification performed.

Characteristics	Overall (N=31) N (%)
Age at Enrollment (Years)	
Mean	14
Median (Range)	14 (8, 17)
Sex	
Female	13 (41.9%)
Male	18 (58.1%)
Race	
Black	16 (51.6%)
White	10 (32.3%)
Other/Unknown	5 (16.1%)
Ethnicity	
Hispanic or Latino	2 (6.5%)
Not Hispanic or Latino	29 (93.5%)
AJCC Stage	
Stage IIB	4 (12.9%)
Stage III	9 (29.0%)
Stage IVA	10 (32.3%)
Stage IVB	6 (19.4%)
Stage IVC	2 (6.5%)

Supplemental Table S6: Details of 20 patients with pre-treatment and post-induction P-EBV samples from Figure 6.

ID	Age at enrollment (Years)	Race	AJCC Stage	pEBV IU/mL Pre-Treatment	pEBV IU/mL Post-Induction	Percent (%) Change from Pre-Treatment to Post-Induction	Total Radiation Dose (Gy)	Post Consolidation Response	First Event	Vital Status
102	16	Black	Stage III	24	75	212.5	70	Stable Disease		Alive
34	12	Black	Stage IVB	313	235	-24.92	61	Partial Response		Alive
44	14	White	Stage III	49	20	-59.18	61	Partial Response		Alive
32	15	Black	Stage IVA	959	158	-83.52	61	Stable Disease	Relapse/Progression	Death
29	17	Unknown	Stage IIB	354	14	-96.05	64	Partial Response		Alive
24	13	Black	Stage IVA	1332	34	-97.45	70	Stable Disease		Alive
90	14	Black	Stage III	2751	59	-97.86	61	Complete Response	Relapse/Progression	Death
33	15	White	Stage IVB	1142	24	-97.9	61	Partial Response		Alive
77	16	Black	Stage IVC	3777	78	-97.93	61	Partial Response	Relapse/Progression	Death
6	14	White	Stage III	615	0	-100	61	Partial Response	Death	Death
16	14	Black	Stage IIB	1180	0	-100	61	Partial Response		Alive
27	13	Black	Stage IVB	6272	0	-100	61	Partial Response		Alive
38	15	Black	Stage IVB	695	0	-100	61	Partial Response		Alive
39	13	White	Stage III	53534	0	-100		No Data		Alive
47	12	White	Stage IVC	249	0	-100	61	Complete Response		Alive
55	14	Black	Stage IVA	870	0	-100	61	Complete Response		Alive
63	15	White	Stage IVB	10426	0	-100	70	Partial Response		Alive
65	14	Unknown	Stage IVB	21459	0	-100	61	Partial Response		Alive
75	8	Black	Stage IVA	247	0	-100		No Data		Alive
97	15	Black	Stage IVA	104	0	-100	70	Partial Response		Alive

Supplemental Table S7 Patient characteristics of 30 patients treated on GPOH with post-induction EBV DNA quantification performed.

Characteristics	Overall (N=30)	P-EBV (N=12)	WB-EBV (N=18)
**Age at enrollment (years) mean**	15.52	14.76	16.03
**Median (range)**	15.51 (9.48-25.21)	12.839 (9.97-25.21)	16.556 (9.48-20.06)
**Sex**			
Female	14	5	9
Male	16	7	9
**Stage of disease**			
I	0	0	0
II	0	0	0
III	6	1	5
IV	24	11	13
**Study**			
NPC-2003	10	10	0
Interim	10	2	8
NPC-2016	10	0	10
**Remission status at end of induction**			
CR	4	3	1
VGPR	7	3	4
PR	18	6	12
SD	0	0	0
PD	0	0	0
No data	1	0	1
**Survival**			
Alive	28	12	16
Died of Disease	1	0	1
Died in CR	1	0	1

Supplemental Table S8: Characteristics of patients from GPOH cohort with post induction P-EBV percent change comparing pre-treatment and post-induction as shown in Figure 6.

Case number	Age at time of diagnosis	Stage of disease	RTH dose	Response after induction	Response after RTH	Response after INF-B	Current status	Pre-treatment p-EBV (Copies/ml)	Post-induction p-EBV (Copies/ml)	Percent change
1	18.8	4	59.4	VGPR	VGPR	VGPR	CR2	110	0	-100
2	11.6	4	59.4	PR	VGPR	CR	CR	135	0	-100
3	12.8	3	54	CR	CR	CR	CR	86	0	-100
4	12.5	4	54	CR	CR	CR	CR	3826	0	-100
5	16.4	4	54	CR	CR	CR	CR	1236	0	-100
6	25.2	4	59.4	PR	PR	CR	CR	38619	34	-99.91
7	13.6	4	59.4	VGPR	VGPR	CR	CR	188	58	-69.14
8	10.8	4	59.4	PR	CR	CR	CR	0	0	No change
9	12.4	4	59.4	VGPR	No data	CR	CR	0	0	No change

Supplemental Figure S2

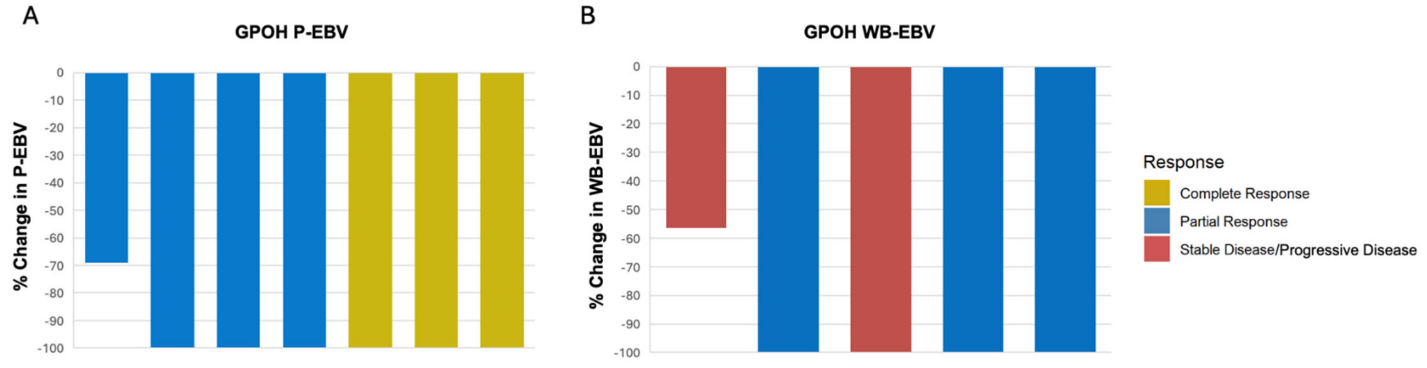

Supplemental Figure S2 Waterfall plot of EBV-DNA percent change post-induction chemotherapy for GPOH cohorts colored by radiological response after chemoradiotherapy. (A) Percent change of EBV-DNA post-induction chemotherapy in NPC patients with positive EBV-DNA pre-treatment levels (GPOH-p-EBV cohort, n=9 (2 negative pre-treatment and post-induction)) (B) Percent change of EBV-DNA post-induction chemotherapy in NPC patients with positive EBV-DNA pre-treatment levels (GPOH-WB-EBV cohort). Yellow = Complete Response (CR) or Very Good Partial Response (VGPR) and Blue = Partial Response (PR), Red = Stable Disease or Progressive Disease at end of Radiation Therapy.

Supplemental Table S9: Characteristics of patients from GPOH cohort with post induction WB-EBV percent change comparing pre-treatment and post-induction as shown in Figure 6.

Case number	Age at time of diagnosis	Stage of disease	RTH dose	Response after induction	Response after RTH	Response after INF-B	Current status	Pre-treatment WB-EBV (IU/mL)	Post-induction WB-EBV (IU/ml)	percent change
1	13.1	4	59.4	PR	PR	VGPR	CR	1982	0	-100
2	16.6	4	61.2	PR	PR	PR	CR	4000	0	-100
3	17.4	4	59.4	PR	SD	SD	SD	515	0	-100
4	18.9	4	59.4	PR	VGPR	CR	CR	1405	0	-100
5	12.7	4	59.4	PR	PD	Not applicable	Died	1118	486	-56.5
6	14.3	4	59.4	PR	CR	CR	CR	0	0	No change
7	15.8	4	59.4	VGPR	Not done	No data	CR	0	0	No change
8	17.4	3	59.4	VGPR	Not done	CR	CR	0	0	No change
9	15.5	4	NID	PR	VGPR	No data	CR	0	0	No change
10	18.4	3	54	CR	VGPR	CR	CR	0	0	No change
11	18.2	3	59.4	VGPR	CR	CR	Died on CR	0	0	No change
12	18.6	3	59.4	PR	PR	CR	CR	0	0	No change
13	18.8	3	59.4	PR	PR	VGPR	CR	0	0	No change
